# 6-Eth­oxy­carbonyl-5,7-dihy­droxy-2,3-dihydro-1*H*-pyrido[3,2,1-*ij*]quinolinium tribromide

**DOI:** 10.1107/S1600536812049276

**Published:** 2012-12-15

**Authors:** Victor B. Rybakov, Svitlana V. Shishkina, Igor V. Ukrainets, Nikolai Yu. Golik, Igor N. Chernenok

**Affiliations:** a Department of Chemistry, Moscow State University, Moscow 119992, Russian Federation; b STC ‘Institute for Single Crystals’, National Academy of Sciences of Ukraine, 60 Lenina Avenue, Kharkiv 61001, Ukraine; c National University of Pharmacy, 4 Blyukhera Street, Kharkiv 61002, Ukraine

## Abstract

In the title salt, C_15_H_16_NO_4_^+.^Br_3_^−^, classical intra­molecular O—H⋯O hydrogen bonds are found, which results in the co-planarity of the ester substituents with the quinolinium residue [C—C—C—O torsion angle = 1.0 (10)°]. The bromine anions are placed on both sides of heterocyclic cation and form Br⋯N contacts of 3.674 (9) and 3.860 (9) Å, which confirms the location of positive charge on the N atom. Non-classical inter­molecular C—H⋯Br inter­actions stabilize the three-dimensional crystal structure. Moreover, anion⋯π inter­actions are noted [Br⋯ring centroid range = 3.367 (9)–3.697 (9) Å]. The partly saturated heterocycle is disordered over two *sofa* conformations with occupancies in the ratio 0.56 (2):0.44 (2).

## Related literature

For general background, see: Ukrainets *et al.* (2004 ▶, 2007 ▶). For chemical bond lengths, see: Bürgi & Dunitz (1994 ▶).
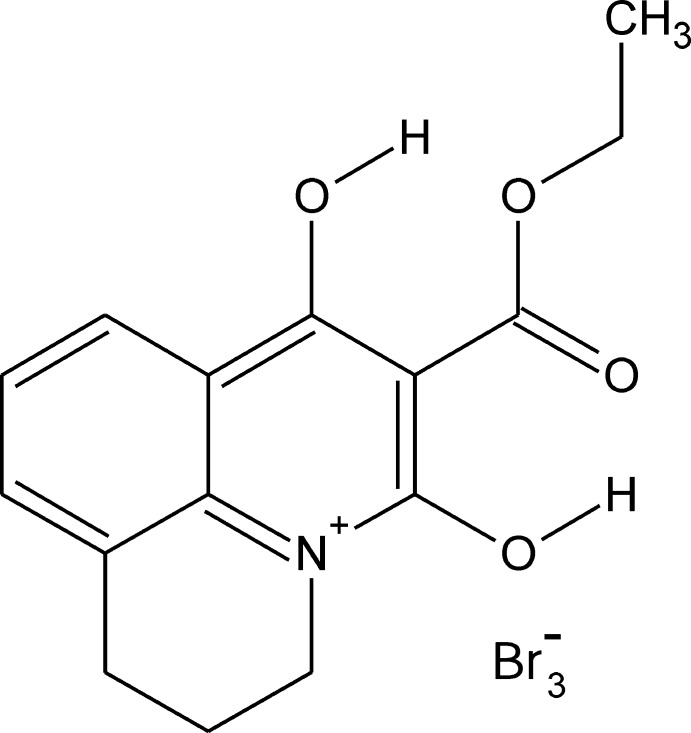


## Experimental

### 

#### Crystal data

C_15_H_16_NO_4_^+^·Br_3_^−^
*M* *_r_* = 513.99
Triclinic, 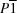

*a* = 7.6491 (8) Å
*b* = 9.1729 (10) Å
*c* = 13.3722 (14) Å
α = 102.355 (9)°
β = 98.777 (9)°
γ = 98.093 (9)°
*V* = 891.06 (17) Å^3^
*Z* = 2
Mo *K*α radiation
μ = 6.81 mm^−1^
*T* = 295 K
0.20 × 0.05 × 0.05 mm

#### Data collection

Agilent Xcalibur-3 CCD diffractometer
Absorption correction: multi-scan (*CrysAlis RED*; Agilent, 2011 ▶) *T*_min_ = 0.343, *T*_max_ = 0.727
10147 measured reflections
5106 independent reflections
1855 reflections with *I* > 2σ(*I*)
*R*_int_ = 0.034

#### Refinement

*R*[*F* ^2^ > 2σ(*F* ^2^)] = 0.068
*wR*(*F* ^2^) = 0.217
*S* = 0.90
5106 reflections
224 parameters
5 restraints
H-atom parameters constrained
Δρ_max_ = 1.02 e Å^−3^
Δρ_min_ = −0.74 e Å^−3^

### 

Data collection: *CrysAlis CCD* (Agilent, 2011 ▶); cell refinement: *CrysAlis RED* (Agilent, 2011 ▶); data reduction: *CrysAlis RED*; program(s) used to solve structure: *SHELXTL* (Sheldrick, 2008 ▶); program(s) used to refine structure: *SHELXTL*; molecular graphics: *ORTEP-3* (Farrugia, 1997 ▶); software used to prepare material for publication: *SHELXTL*.

## Supplementary Material

Crystal structure: contains datablock(s) I, global. DOI: 10.1107/S1600536812049276/aa2080sup1.cif

Structure factors: contains datablock(s) I. DOI: 10.1107/S1600536812049276/aa2080Isup2.hkl

Supplementary material file. DOI: 10.1107/S1600536812049276/aa2080Isup3.cml

Additional supplementary materials:  crystallographic information; 3D view; checkCIF report

## Figures and Tables

**Table 1:** Hydrogen-bond geometry (Å, °)

*D*—H⋯*A*	*D*—H	H⋯*A*	*D*⋯*A*	*D*—H⋯*A*
O2—H2⋯O4	0.82	1.93	2.631 (7)	142
O1—H1⋯O3	0.82	1.73	2.459 (10)	147
C3—H3⋯Br1^i^	0.93	2.90	3.810 (9)	168
C4—H4⋯Br2^ii^	0.93	3.06	3.846 (9)	143
C10—H10*B*⋯Br2	0.97	2.99	3.936 (8)	166
C10—H10*C*⋯Br4^iii^	0.97	2.92	3.752 (8)	144
C11*A*—H11*B*⋯Br4^iii^	0.97	3.02	3.797 (17)	138

Symmetry codes: (i) 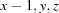; (ii) 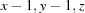; (iii) 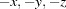.

**Fig. 1.**
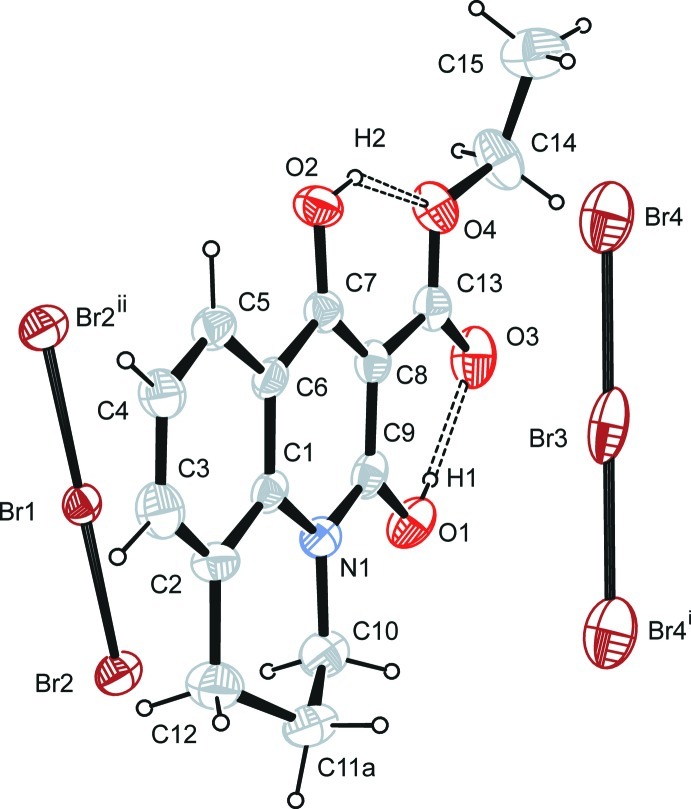
View of the title compound with the atom numbering scheme. The displacement ellipsoids are drawn at the 15% probability level. H atoms are presented as a small spheres of arbitrary radius. Intramolecular hydrogen bonds are indicated by dashed lines. Only major moiety of disorder group [s.o.f. = 0.56 (2)] are presented. Symmetry codes: (i) -x + 1, -y + 1, -z + 1; (ii) -x, -y, -z.

## supplementary crystallographic information

### Comment

Bromination of alkyl 1-*R*-4-hydroxy-2-oxo-1,2-dihydroquinoline-3-carboxylates (R = H, alkyl, phenyl) by molecular bromine in the environment of acetic acid can pass on two directions (Ukrainets *et al.*, 2004, 2007). However, the reaction of ethyl 7-hydroxy-5-oxo-2,3-dihydro-1*H*,5*H*-pyrido[3,2,1-*ij*]quinoline-6-carboxylate with bromine results in the 6-ethyloxycarbonyl-5,7-dihydroxy-2,3-dihydro-1*H*-pyrido[3,2,1-*ij*]quinolinium tribromide, I.

Two tribromide anions are located in special positions, the coordinates of the central atoms coincide with the center of symmetry so the asymmetric part of unit cell contains one cation and two halves of anions. The positive charge of the cation is located on the N atom which is bonded with three atoms and the N1═C9 bond (1.335 (6) Å) has double character (the mean value for C*sp*^2^═N bond is 1.329 (1) Å (Bürgi & Dunitz, 1994)).

The partly saturated heterocycle is disordered over two sofa conformations (*A* and *B*) with population in the ratio 0.56 (2)/0.44 (2). The deviations of the C11 atom from main plane C1/C2/N1/C10/C12 are -0.57 (1) and 0.59 (1) Å for conformers *A* and *B*, respectively. The ester substituent is coplanar to the planar fragment of tricycle (C9—C8—C13—O3 torsion angle is 1.0 (10)°) owing to the formation of the strong intramolecular hydrogen bonds O1—H1···O3 and O2—H2···O4 (Table 1). The formation of hydrogen bonds causes the elongation of the C13═O3 (1.243 (10) Å) and C7═C8 (1.376 (9) Å) bonds (the mean values are 1.210 (1) Å and 1.326 (1) Å, respectively) and the shortening of the C9—O1 (1.307 (8) Å), C7—O2 (1.299 (8) Å) bonds (the mean value is 1.333 (1) Å). The methylene atom from ethyl group of the substituent has *ap*-orientation relative to the C8—C13 bond and is turned relative to the C13—O4 bond (the C14—O4—C13—C8 and C13—O4—C14—C15 torsion angles are 175.7 (6)° and -155.4 (8)°, respectively).

### Experimental

A solution of anhydrous bromine (0.52 ml, 0.01 mol) in anhydrous acetic acid (5 ml) was added with vigorous stirring to a solution of the ethyl 7-hydroxy-5-oxo-2,3-dihydro-1*H*,5*H*-pyrido[3,2,1-*ij*]quinoline-6-carboxylate (2.73 g, 0.01 mol) in anhydrous acetic acid (20 ml). A light-yellow precipitate was formed immediately. The crystals of I were filtered off, washed with acetic acid and dried to give the product (2.26 g, 44%); m.p. 360–362 K.

### Refinement

The restrictions on the bond length of the ethyl group of the ester substituent and bond lengths in disordered fragment (1.54 Å) were applied. All H atoms were located from electron-density difference maps and were refined in the riding-motion approximation with *U*_iso_(H) constrained to be 1.5 times *U*_eq_ of the carrier atom for the methyl and hydroxyl groups and 1.2 times *U*_eq_ of the carrier atom for the other atoms.

### Crystal data

**Table:**

C_15_H_16_NO_4_^+^·Br_3_^−^	*Z* = 2
*M**_r_* = 513.99	*F*(000) = 500
Triclinic, *P*1	*D*_x_ = 1.916 Mg m^−^^3^
Hall symbol: -P 1	Melting point = 360–362 K
*a* = 7.6491 (8) Å	Mo *K*α radiation, λ = 0.71073 Å
*b* = 9.1729 (10) Å	Cell parameters from 1363 reflections
*c* = 13.3722 (14) Å	θ = 3.1–32.0°
α = 102.355 (9)°	µ = 6.81 mm^−^^1^
β = 98.777 (9)°	*T* = 295 K
γ = 98.093 (9)°	Rod, light yellow
*V* = 891.06 (17) Å^3^	0.20 × 0.05 × 0.05 mm

### Data collection

**Table:**

Agilent Xcalibur-3 CCD diffractometer	5106 independent reflections
Radiation source: Enhance (Mo) X-Ray Source	1855 reflections with *I* > 2σ(*I*)
Graphite monochromator	*R*_int_ = 0.034
Detector resolution: 16.1827 pixels mm^-1^	θ_max_ = 30.0°, θ_min_ = 3.1°
ω scans	*h* = −10→10
Absorption correction: multi-scan (*CrysAlis RED*; Agilent, 2011)	*k* = −12→12
*T*_min_ = 0.343, *T*_max_ = 0.727	*l* = −18→18
10147 measured reflections	

### Refinement

**Table:**

Refinement on *F*^2^	Primary atom site location: structure-invariant direct methods
Least-squares matrix: full	Secondary atom site location: difference Fourier map
*R*[*F*^2^ > 2σ(*F*^2^)] = 0.068	Hydrogen site location: difference Fourier map
*wR*(*F*^2^) = 0.217	H-atom parameters constrained
*S* = 0.90	*w* = 1/[σ^2^(*F*_o_^2^) + (0.108*P*)^2^] where *P* = (*F*_o_^2^ + 2*F*_c_^2^)/3
5106 reflections	(Δ/σ)_max_ < 0.001
224 parameters	Δρ_max_ = 1.02 e Å^−^^3^
5 restraints	Δρ_min_ = −0.74 e Å^−^^3^

### Special details

**Table:**

Experimental. Absorption correction: *CrysAlis RED* (Agilent Technologies, 2011). Empirical absorption correction using spherical harmonics, implemented in *SCALE3 ABSPACK* scaling algorithm.
Geometry. All s.u.'s (except the s.u. in the dihedral angle between two l.s. planes) are estimated using the full covariance matrix. The cell s.u.'s are taken into account individually in the estimation of s.u.'s in distances, angles and torsion angles; correlations between s.u.'s in cell parameters are only used when they are defined by crystal symmetry. An approximate (isotropic) treatment of cell s.u.'s is used for estimating s.u.'s involving l.s. planes.
Refinement. Refinement of *F*^2^ against ALL reflections. The weighted *R*-factor w*R*and goodness of fit *S* are based on *F*^2^, conventional *R*-factors *R* are based on *F*, with *F* set to zero for negative *F*^2^. The threshold expression of *F*^2^ > 2σ(*F*^2^) is used only for calculating *R*-factors(gt) *etc*. and is not relevant to the choice of reflections for refinement. *R*-factors based on *F*^2^ are statistically about twice as large as those based on *F*, and *R*-factors based on ALL data will be even larger.

### Fractional atomic coordinates and isotropic or equivalent isotropic displacement parameters (Å^2^)

**Table:**

	*x*	*y*	*z*	*U*_iso_*/*U*_eq_	Occ. (<1)
Br1	0.5000	0.5000	0.5000	0.0803 (3)	
Br2	0.56781 (11)	0.74499 (9)	0.44305 (6)	0.1062 (4)	
Br3	0.0000	0.0000	0.0000	0.1391 (6)	
Br4	0.06301 (17)	−0.24813 (17)	0.03287 (9)	0.1666 (6)	
N1	0.1821 (7)	0.3314 (6)	0.2533 (4)	0.0753 (14)	
O1	0.4153 (7)	0.3418 (7)	0.1698 (5)	0.1109 (17)	
H1	0.4995	0.2986	0.1580	0.166*	
O2	0.2437 (8)	−0.0849 (6)	0.3048 (4)	0.0989 (14)	
H2	0.3201	−0.1217	0.2760	0.148*	
O3	0.5774 (8)	0.1288 (8)	0.1341 (4)	0.131 (2)	
O4	0.5025 (7)	−0.0768 (7)	0.1961 (4)	0.1052 (16)	
C1	0.0665 (9)	0.2577 (8)	0.3053 (4)	0.0726 (17)	
C2	−0.0723 (9)	0.3279 (8)	0.3413 (6)	0.0848 (19)	
C3	−0.1792 (11)	0.2510 (11)	0.3935 (6)	0.108 (2)	
H3	−0.2679	0.2972	0.4209	0.129*	
C4	−0.1624 (11)	0.1110 (10)	0.4074 (6)	0.095 (2)	
H4	−0.2438	0.0610	0.4394	0.114*	
C5	−0.0296 (10)	0.0449 (8)	0.3754 (5)	0.0853 (19)	
H5	−0.0136	−0.0483	0.3888	0.102*	
C6	0.0867 (9)	0.1169 (8)	0.3211 (4)	0.0747 (17)	
C7	0.2325 (9)	0.0461 (8)	0.2845 (5)	0.0774 (17)	
C8	0.3425 (9)	0.1203 (8)	0.2317 (5)	0.0772 (17)	
C9	0.3130 (10)	0.2649 (9)	0.2176 (5)	0.085 (2)	
C10	0.1679 (10)	0.4818 (9)	0.2325 (7)	0.110 (3)	
H10A	0.1998	0.4849	0.1654	0.132*	0.56 (2)
H10B	0.2520	0.5597	0.2855	0.132*	0.56 (2)
H10C	0.1017	0.4691	0.1624	0.132*	0.44 (2)
H10D	0.2871	0.5389	0.2378	0.132*	0.44 (2)
C11A	−0.0241 (14)	0.515 (2)	0.2325 (11)	0.113 (7)	0.56 (2)
H11A	−0.0223	0.6214	0.2353	0.136*	0.56 (2)
H11B	−0.1011	0.4569	0.1675	0.136*	0.56 (2)
C11B	0.0704 (18)	0.5691 (14)	0.3120 (14)	0.117 (9)	0.44 (2)
H11C	0.1506	0.6019	0.3793	0.141*	0.44 (2)
H11D	0.0431	0.6591	0.2904	0.141*	0.44 (2)
C12	−0.1052 (12)	0.4752 (10)	0.3240 (7)	0.126 (3)	
H12A	−0.0535	0.5536	0.3869	0.151*	0.56 (2)
H12B	−0.2338	0.4734	0.3099	0.151*	0.56 (2)
H12C	−0.1499	0.5296	0.3825	0.151*	0.44 (2)
H12D	−0.1956	0.4597	0.2615	0.151*	0.44 (2)
C13	0.4866 (11)	0.0567 (11)	0.1831 (5)	0.092 (2)	
C14	0.6366 (11)	−0.1428 (11)	0.1409 (8)	0.140 (4)	
H14A	0.7564	−0.1079	0.1829	0.167*	
H14B	0.6352	−0.1137	0.0752	0.167*	
C15	0.5833 (15)	−0.3161 (11)	0.1218 (10)	0.173 (5)	
H15A	0.6558	−0.3649	0.0772	0.260*	
H15B	0.4588	−0.3470	0.0893	0.260*	
H15C	0.6020	−0.3444	0.1872	0.260*	

### Atomic displacement parameters (Å^2^)

**Table:**

	*U*^11^	*U*^22^	*U*^33^	*U*^12^	*U*^13^	*U*^23^
Br1	0.0730 (5)	0.0913 (7)	0.0716 (5)	0.0189 (5)	0.0020 (4)	0.0143 (5)
Br2	0.1086 (6)	0.0994 (7)	0.1052 (6)	0.0040 (5)	0.0010 (4)	0.0359 (5)
Br3	0.0870 (7)	0.2340 (17)	0.0652 (6)	−0.0272 (9)	−0.0029 (5)	0.0175 (7)
Br4	0.1423 (10)	0.2122 (13)	0.1221 (8)	−0.0008 (9)	0.0014 (6)	0.0283 (8)
N1	0.071 (3)	0.071 (4)	0.077 (3)	−0.003 (3)	0.004 (3)	0.019 (3)
O1	0.098 (4)	0.133 (5)	0.108 (4)	−0.009 (3)	0.022 (3)	0.061 (4)
O2	0.112 (4)	0.082 (3)	0.116 (4)	0.034 (3)	0.040 (3)	0.028 (3)
O3	0.107 (4)	0.179 (6)	0.104 (4)	−0.004 (4)	0.050 (3)	0.027 (4)
O4	0.083 (3)	0.110 (4)	0.114 (4)	0.011 (3)	0.031 (3)	0.002 (3)
C1	0.076 (4)	0.076 (4)	0.055 (3)	−0.003 (3)	−0.006 (3)	0.017 (3)
C2	0.084 (4)	0.082 (5)	0.099 (5)	0.026 (4)	0.020 (4)	0.034 (4)
C3	0.095 (5)	0.125 (7)	0.103 (5)	0.021 (5)	0.035 (4)	0.016 (5)
C4	0.096 (5)	0.104 (6)	0.095 (5)	0.029 (5)	0.040 (4)	0.024 (4)
C5	0.097 (5)	0.083 (5)	0.081 (4)	0.009 (4)	0.023 (4)	0.030 (4)
C6	0.086 (4)	0.080 (5)	0.052 (3)	0.007 (4)	−0.002 (3)	0.019 (3)
C7	0.080 (4)	0.076 (5)	0.069 (4)	0.007 (4)	0.008 (3)	0.012 (3)
C8	0.076 (4)	0.084 (5)	0.062 (3)	0.002 (4)	0.006 (3)	0.010 (3)
C9	0.085 (5)	0.096 (6)	0.059 (3)	−0.017 (4)	−0.002 (3)	0.019 (4)
C10	0.113 (6)	0.086 (6)	0.113 (6)	−0.014 (5)	−0.012 (5)	0.027 (5)
C11A	0.115 (13)	0.103 (12)	0.127 (14)	0.022 (10)	0.004 (10)	0.052 (11)
C11B	0.111 (16)	0.081 (14)	0.17 (2)	0.015 (11)	0.018 (16)	0.060 (14)
C12	0.141 (8)	0.099 (6)	0.146 (8)	0.029 (6)	0.034 (6)	0.038 (6)
C13	0.092 (5)	0.102 (6)	0.072 (4)	0.014 (5)	0.008 (4)	0.009 (4)
C14	0.097 (6)	0.154 (10)	0.151 (8)	0.033 (6)	0.043 (6)	−0.019 (7)
C15	0.176 (11)	0.169 (12)	0.232 (13)	0.074 (10)	0.106 (10)	0.096 (11)

### Geometric parameters (Å, º)

**Table:**

Br1—Br2^i^	2.5346 (8)	C7—C8	1.376 (9)
Br1—Br2	2.5346 (8)	C8—C9	1.423 (10)
Br3—Br4^ii^	2.5057 (16)	C8—C13	1.489 (11)
Br3—Br4	2.5057 (16)	C10—C11A	1.5398 (10)
N1—C9	1.340 (9)	C10—C11B	1.5398 (10)
N1—C1	1.394 (8)	C10—H10A	0.9700
N1—C10	1.479 (9)	C10—H10B	0.9700
O1—C9	1.307 (8)	C10—H10C	0.9700
O1—H1	0.8200	C10—H10D	0.9700
O2—C7	1.299 (8)	C11A—C12	1.5396 (10)
O2—H2	0.8200	C11A—H11A	0.9700
O3—C13	1.243 (10)	C11A—H11B	0.9700
O4—C13	1.292 (9)	C11B—C12	1.5397 (10)
O4—C14	1.476 (9)	C11B—H11C	0.9700
C1—C6	1.378 (9)	C11B—H11D	0.9700
C1—C2	1.411 (10)	C12—H12A	0.9700
C2—C3	1.370 (10)	C12—H12B	0.9700
C2—C12	1.467 (11)	C12—H12C	0.9700
C3—C4	1.358 (10)	C12—H12D	0.9700
C3—H3	0.9300	C14—C15	1.5395 (10)
C4—C5	1.334 (10)	C14—H14A	0.9700
C4—H4	0.9300	C14—H14B	0.9700
C5—C6	1.411 (9)	C15—H15A	0.9600
C5—H5	0.9300	C15—H15B	0.9600
C6—C7	1.462 (10)	C15—H15C	0.9600
			
Br2^i^—Br1—Br2	180.0	C11B—C10—H10D	109.7
Br4^ii^—Br3—Br4	180.00 (8)	H10A—C10—H10D	66.6
C9—N1—C1	120.0 (6)	H10C—C10—H10D	108.2
C9—N1—C10	116.4 (6)	C12—C11A—C10	113.6 (7)
C1—N1—C10	123.6 (6)	C12—C11A—H11A	108.9
C9—O1—H1	109.5	C10—C11A—H11A	108.9
C7—O2—H2	109.5	C12—C11A—H11B	108.9
C13—O4—C14	112.8 (7)	C10—C11A—H11B	108.9
C6—C1—N1	120.4 (6)	H11A—C11A—H11B	107.7
C6—C1—C2	120.1 (6)	C12—C11B—C10	113.6 (7)
N1—C1—C2	119.5 (6)	C12—C11B—H11C	108.9
C3—C2—C1	116.7 (7)	C10—C11B—H11C	108.9
C3—C2—C12	120.4 (8)	C12—C11B—H11D	108.9
C1—C2—C12	122.9 (6)	C10—C11B—H11D	108.9
C4—C3—C2	123.5 (8)	H11C—C11B—H11D	107.7
C4—C3—H3	118.3	C2—C12—C11A	112.3 (9)
C2—C3—H3	118.3	C2—C12—C11B	109.9 (10)
C5—C4—C3	120.2 (7)	C2—C12—H12A	109.1
C5—C4—H4	119.9	C11A—C12—H12A	109.1
C3—C4—H4	119.9	C11B—C12—H12A	68.9
C4—C5—C6	119.7 (7)	C2—C12—H12B	109.1
C4—C5—H5	120.1	C11A—C12—H12B	109.1
C6—C5—H5	120.1	C11B—C12—H12B	139.4
C1—C6—C5	119.7 (7)	H12A—C12—H12B	107.9
C1—C6—C7	119.5 (6)	C2—C12—H12C	109.7
C5—C6—C7	120.7 (7)	C11A—C12—H12C	136.4
O2—C7—C8	126.0 (7)	C11B—C12—H12C	109.7
O2—C7—C6	115.3 (6)	H12B—C12—H12C	66.2
C8—C7—C6	118.7 (7)	C2—C12—H12D	109.7
C7—C8—C9	118.7 (7)	C11A—C12—H12D	67.7
C7—C8—C13	124.5 (7)	C11B—C12—H12D	109.7
C9—C8—C13	116.7 (7)	H12A—C12—H12D	138.7
O1—C9—N1	116.0 (7)	H12B—C12—H12D	45.0
O1—C9—C8	121.3 (8)	H12C—C12—H12D	108.2
N1—C9—C8	122.6 (6)	O3—C13—O4	125.5 (8)
N1—C10—C11A	111.1 (8)	O3—C13—C8	120.9 (9)
N1—C10—C11B	109.7 (9)	O4—C13—C8	113.6 (7)
N1—C10—H10A	109.4	O4—C14—C15	106.3 (8)
C11A—C10—H10A	109.4	O4—C14—H14A	110.5
C11B—C10—H10A	139.2	C15—C14—H14A	110.5
N1—C10—H10B	109.4	O4—C14—H14B	110.5
C11A—C10—H10B	109.4	C15—C14—H14B	110.5
C11B—C10—H10B	68.8	H14A—C14—H14B	108.7
H10A—C10—H10B	108.0	C14—C15—H15A	109.5
N1—C10—H10C	109.7	C14—C15—H15B	109.5
C11A—C10—H10C	68.1	H15A—C15—H15B	109.5
C11B—C10—H10C	109.7	C14—C15—H15C	109.5
H10B—C10—H10C	138.4	H15A—C15—H15C	109.5
N1—C10—H10D	109.7	H15B—C15—H15C	109.5
C11A—C10—H10D	137.5		
			
C9—N1—C1—C6	0.6 (8)	C10—N1—C9—C8	−179.9 (6)
C10—N1—C1—C6	179.4 (5)	C7—C8—C9—O1	178.7 (6)
C9—N1—C1—C2	−179.1 (6)	C13—C8—C9—O1	−4.2 (9)
C10—N1—C1—C2	−0.3 (9)	C7—C8—C9—N1	−0.2 (9)
C6—C1—C2—C3	1.5 (9)	C13—C8—C9—N1	176.9 (6)
N1—C1—C2—C3	−178.8 (6)	C9—N1—C10—C11A	155.4 (8)
C6—C1—C2—C12	−177.9 (7)	C1—N1—C10—C11A	−23.5 (10)
N1—C1—C2—C12	1.8 (10)	C9—N1—C10—C11B	−157.4 (8)
C1—C2—C3—C4	−3.0 (12)	C1—N1—C10—C11B	23.7 (10)
C12—C2—C3—C4	176.4 (7)	N1—C10—C11A—C12	45.2 (15)
C2—C3—C4—C5	4.4 (13)	C11B—C10—C11A—C12	−51.8 (8)
C3—C4—C5—C6	−4.0 (11)	N1—C10—C11B—C12	−48.6 (16)
N1—C1—C6—C5	178.9 (5)	C11A—C10—C11B—C12	51.8 (8)
C2—C1—C6—C5	−1.4 (9)	C3—C2—C12—C11A	−158.5 (10)
N1—C1—C6—C7	1.0 (8)	C1—C2—C12—C11A	20.9 (12)
C2—C1—C6—C7	−179.3 (6)	C3—C2—C12—C11B	154.1 (9)
C4—C5—C6—C1	2.6 (10)	C1—C2—C12—C11B	−26.5 (11)
C4—C5—C6—C7	−179.5 (7)	C10—C11A—C12—C2	−44.3 (16)
C1—C6—C7—O2	178.5 (5)	C10—C11A—C12—C11B	51.8 (8)
C5—C6—C7—O2	0.6 (9)	C10—C11B—C12—C2	50.2 (17)
C1—C6—C7—C8	−2.2 (8)	C10—C11B—C12—C11A	−51.8 (8)
C5—C6—C7—C8	179.9 (6)	C14—O4—C13—O3	−3.2 (11)
O2—C7—C8—C9	−179.0 (6)	C14—O4—C13—C8	175.7 (6)
C6—C7—C8—C9	1.8 (9)	C7—C8—C13—O3	178.0 (7)
O2—C7—C8—C13	4.1 (11)	C9—C8—C13—O3	1.0 (10)
C6—C7—C8—C13	−175.1 (6)	C7—C8—C13—O4	−1.0 (10)
C1—N1—C9—O1	180.0 (5)	C9—C8—C13—O4	−177.9 (6)
C10—N1—C9—O1	1.1 (8)	C13—O4—C14—C15	−155.4 (8)
C1—N1—C9—C8	−1.0 (8)		

Symmetry codes: (i) −*x*+1, −*y*+1, −*z*+1; (ii) −*x*, −*y*, −*z*.

### Hydrogen-bond geometry (Å, º)

**Table:**

*D*—H···*A*	*D*—H	H···*A*	*D*···*A*	*D*—H···*A*
O2—H2···O4	0.82	1.93	2.631 (7)	142
O1—H1···O3	0.82	1.73	2.459 (10)	147
C3—H3···Br1^iii^	0.93	2.90	3.810 (9)	168
C4—H4···Br2^iv^	0.93	3.06	3.846 (9)	143
C10—H10*B*···Br2	0.97	2.99	3.936 (8)	166
C10—H10*C*···Br4^ii^	0.97	2.92	3.752 (8)	144
C11*A*—H11*B*···Br4^ii^	0.97	3.02	3.797 (17)	138

Symmetry codes: (ii) −*x*, −*y*, −*z*; (iii) *x*−1, *y*, *z*; (iv) *x*−1, *y*−1, *z*.
